# Editorial: Editors’ showcase: hepatology

**DOI:** 10.3389/fgstr.2023.1198142

**Published:** 2023-04-21

**Authors:** Nipun Verma, Ashwani K. Singal

**Affiliations:** ^1^ Department of Hepatology, Post Graduate Institute of Medical Education and Research, Chandigarh, India; ^2^ University of South Dakota Sanford School of Medicine, University of South Dakota, Vermillion, SD, United States; ^3^ Department of Transplant Hepatology, Avera McKennan Hospital, Sioux Falls, SD, United States; ^4^ Health Research Science, Veterans Affairs (VA) Medical Center, Sioux Falls, SD, United States

**Keywords:** liver - fibrosis and - cirrhosis, HCC, transplant, DILI, advancement

There have been significant innovations and advances in hepatology during the recent years. Frontiers and the editorial team of Liver section of their Gastroenterology journal needs to be congratulated for putting together this Research Topic “*Editors Showcase: Hepatology*”. The Research Topic has included a total of nine articles which are summarized below:

Li et al. in this Research Topic described a case of embryonal rhabdomyosarcoma of the liver in a 7-year-old boy, a rare malignant tumor. The patient presented with abnormal levels of serum tumor markers and liver function, and imaging revealed a large mass in the left lobe of the liver. Although the patient was initially misdiagnosed on percutaneous liver biopsy, the final diagnosis was established on postoperative histopathology and immunohistochemical staining by desmin and myogenin. The case highlights the importance of accurate diagnosis and the potential benefits of a neoadjuvant chemotherapy, comprehensive treatment and follow up care for patients with rhabdomyosarcoma.

Ma et al. described the impact of hepatic hydrothorax (HH) on the prognosis of patients with decompensated cirrhosis, a complication in patients with decompensated cirrhosis with a significant negative impact on quality of life. Impact of HH on the patient survival is not entirely clear, a topic which is well addressed in this study, which found that HH is associated with higher mortality in patients with decompensated cirrhosis. This study highlights the need for early recognition and management of HH in patients with decompensated cirrhosis to improve their long-term prognosis.

Wu et al. described a protocol for the Kupffer phase whole liver scan for detecting liver metastases, which can significantly impact the treatment options for cancer patients. This is a self-controlled, blind map-reading, single-center, prospective trial that aims to determine the number, size, location, and diagnosis of metastatic lesions using Sonazoid-contrast enhanced ultrasound (CEUS), a technique to improve the detection of liver lesions vs. a traditional contrast CEUS. The results of this study could have significant implications for the diagnosis and treatment of liver metastases, and avoiding unnecessary specific imaging such as contrast enhanced CT scans.

Pan et al. in a case report highlighted the importance of timely diagnosis and intervention in patients with spontaneous rupture of hepatic hemangioma, a congenital vascular malformation that usually remains asymptomatic, but spontaneous rupture can occur rarely, which can lead to life-threatening complications. Dynamic contrast-enhanced CT scanning, particularly triple-phase CT with delayed imaging, is recommended for accurate diagnosis. Emergency surgery is often necessary to avoid further complications and ensure patient recovery. This case report underscores the awareness of rare but potentially life-threatening complication of rupture, and early diagnosis for successful patient outcome.

Zhao et al. describe a case report highlighting complexity of liver transplantation for large benign hepatic masses, a rare indication for liver transplantation. The patient in this report had undergone various percutaneous operations and open surgeries, resulting in severe perihepatic adhesions, which made the surgery even more challenging. The case underscores the importance of careful patient selection, thorough preoperative evaluation, and specialized surgical expertise in performing liver transplantation for large benign hepatic masses.

The study by Cheng et al. highlights the importance of molecular genetic findings of ATP-binding cassette subfamily B member 4 (ABCB4) and MDR3 immunohistochemistry in diagnosing progressive familial intrahepatic cholestasis type 3 (PFIC3), a rare genetic disorder that can lead to severe liver damage. The identification of a novel mutation of ABCB4 in these patients provides important insights into the pathogenesis of this disorder. This study underscores the importance of a multidisciplinary approach to the diagnosis and management of rare liver disorders, and emphasizes the critical role of molecular genetic testing and histopathological analysis in improving diagnostic accuracy.

Drug-induced liver injury (DILI) is a complex and multifactorial disease that remains challenging to diagnose and prevent in clinical practice. Recent research has highlighted the role of gut microbiota dysbiosis in the development of antibiotic associated DILI. Antibiotics can affect the structure and diversity of gut microbiota, leading to changes in metabolites and a reduction in the efficacy of hepatoprotective agents. Follow-up with liver function examination is essential during the administration of drugs that affect intestinal microorganisms, especially in patients on a high-fat diet. The review by Fu et al. provides valuable insights into the potential prevention and review therapeutic strategies in the management of this challenging disease.

Lin et al. in this Research Topic analyzed the clinical information of 539 patients with primary hepatic neuroendocrine tumors (PHNETs), and established an assessment model for prognosis. They found that surgical treatment and the number of primary malignancies were independent protective factors for PHNETs, and developed a competing risk nomogram with high accuracy in predicting disease-specific survival. This nomogram may help clinicians develop personalized treatment strategies for patients with PHNETs, highlighting the importance of individualized approaches in rare diseases with limited research.

The combination of hepatic arterial infusion chemotherapy (HAIC) with programmed cell death protein-1 (PD-1) antibody and lenvatinib showed a promise as an effective treatment for advanced hepatocellular carcinoma (HCC), according to a retrospective study by Xu et al. The study evaluated 61 patients who underwent the treatment at the Second Affiliated Hospital of Nanchang University and found an objective response rate (ORR) of 36.1% (RECIST 1.1)/57.4% (mRECIST) and a disease control rate (DCR) of 82.0%. The median progression-free survival (PFS) was 6.0 months, with the most common treatment-related adverse events (TRAEs) being neutropenia, abdominal pain, and aspartate aminotransferase increase. The study suggests that HAIC with PD-1 antibody and Lenvatinib is a viable treatment option for advanced HCC.

Overall, this Research Topic covers some of the important aspects in hepatology, and provides comprehensive information for clinicians and researchers. I as the editor-in-chief and the editorial team of hepatology section of Frontiers in gastroenterology believe and hope that this endeavor of ours will be of extreme help the hepatologists and physicians in general in their respective practices.

## Author contributions

The article was conceived and written by NV. Both the authors reviewed the article and approved the final version before submission.

